# Retraction: Specific expression of lncRNA RP13-650J16.1 and TCONS_00023979 in prostate cancer

**DOI:** 10.1042/BSR-2017-1571_RET

**Published:** 2024-02-12

**Keywords:** DU145, prostate cancer, RP13-650J16.1, TCONS_00023979

This article is being retracted from *Bioscience Reports* at the request of the Editor-in-Chief and the Editorial Board following receipt of a notification from a reader, alerting the Editorial Board to a duplicated Western blot in Figure 2B, incorrect primers for the qPCR and regions of duplication in Figure 6A and 6B.

The authors have been contacted in regard to the issues raised and have responded to the concerns on PubPeer. The authors wished to correct the article however, the Editorial Board stand by the decision to retract given the concerns with original data presented in Figures 6A and 6B. The authors state that the Western blot in Figure 2B was misplaced, and that the wrong selection of Figure 6 resulted in the regions of duplication in Figure 6A and 6B. Following discussions with the Editorial Office, the authors agreed to the retraction.

